# Interdisciplinary social needs response team: A community case study in social needs targeted care during the COVID-19 pandemic and beyond

**DOI:** 10.3389/fpubh.2022.789396

**Published:** 2022-11-09

**Authors:** Isabelle Mullen, Melanie Agnes Mariano, Jaya Aysola

**Affiliations:** ^1^Perelman School of Medicine, University of Pennsylvania, Philadelphia, PA, United States; ^2^School of Nursing, University of Pennsylvania, Philadelphia, PA, United States; ^3^Center for Health Equity Advancement, Penn Medicine, Philadelphia, PA, United States

**Keywords:** social needs targeted care, health equity, COVID-19, interdisciplinary, health care services, health systems

## Abstract

This case study illustrates the role and value of a social needs response team during times of crisis and beyond. The COVID-19 pandemic resulted in two simultaneous crises—the infectious disease crisis and the socioeconomic crisis. Unemployment and lost wages, housing and food insecurity, and increased childcare needs are just a few examples of the socioeconomic needs that skyrocketed during the COVID-19 pandemic. At the start of the pandemic, the University of Pennsylvania Health System (UPHS) formed an interdisciplinary team of physicians, social workers, nurse practitioners and students of these professions to reimagine social needs screening in a way that could reach people during the pandemic and provide sustainable support for individual's evolving social needs. The Social Needs Response Team (SNRT) at UPHS utilized various secure platforms to keep members of the team connected with each other and their patients. Orientations for participating students included training on how to employ principles of crisis intervention theory, empathetic inquiry, and patient-led and family-centered care to best uncover and serve the needs of their patients. Alongside the illustrative case study, this piece details guiding principles and concepts that are essential to integrating social needs targeted care.

## Introduction: Case presentation

Mr. R, a 50-year-old man, balances working for a rideshare company with raising three children and applying for U.S. citizenship. His problem list would have included food insecurity, housing instability, health insurance needs, and lack of access to hot water. However, Mr. R never presented to us; instead, we spoke with Ms. R.

Ms. R, a 19-year-old woman who tested positive for COVID-19 and was identified by the Contact Tracing Team, was referred to us, the Social Needs Response Team (SNRT), after she screened positive for needing housing and food access assistance. While screening for any additional unmet needs, we asked if she needed assistance applying for unemployment. She said her father would have to answer that. After finishing the screener, she handed the phone to her father, Mr. R, to discuss the unanswered questions. With the patient's permission, we reviewed her answers with her father. His stress echoed through the phone as he detailed lost wages resulting from his own COVID-19 diagnosis (Mr. R and his three children all had COVID-19), food insecurity, late bill payments, and his landlord's attempt to evict them.

The COVID-19 pandemic has underscored the inadequacy of resources to address unmet basic needs such as these. At the start of the pandemic in the United States, unemployment rates rose sharply from 3.5% in February 2020 to its peak at 14.8% in April 2020. This is the highest unemployment rate since data began being collected in 1948. As of July 2021, unemployment remained at 5.4% (1). Furthermore, job losses have been concentrated in lower income households, with the job loss rate in low-wage industries as high as triple that of losses in high-wage industries. This has led to an increase in food insecurity, which persists today. As of August 2021, 9% of responding adults in the Census Bureau's Household Pulse survey were still experiencing food insecurity, an increase from 3.4% in 2019 surveys. Respondents also reported difficulty affording rent and household expenses (2).

The vast unmet social needs unearthed by COVID-19 and the then-limited interactions with patients posed a unique challenge to healthcare providers hoping to identify and address patient's needs. The Social Needs Response Team (SNRT) is an interdisciplinary, virtual team designed to deliver social needs-targeted care (3). Such care is patient-led, family-centered, applies empathetic inquiry, and is informed by crisis intervention theory.

## Background and rationale

This case study explores how the Social Needs Response Team employs social needs-targeted care to screen for and address unmet social needs during the COVID-19 pandemic.

Social needs-targeted care, unlike social needs-informed care, views social needs assessments as integral to health rather than ancillary. In contrast, social needs-informed care views social needs assessments as information that may be modified to improve access to or quality of healthcare for a patient (such as the need for transportation to get to an appointment). Social needs-targeted care, by contrast, seeks to directly address social needs (such as housing instability or income insecurity) regardless of its immediate impact on healthcare (4).

Mounting evidence demonstrates the impact of social needs on health care use, cost, and quality, leading federal agencies, and national medical societies to call for health care organizations to conduct screenings of social needs. Yet a recent survey of 2,190 physicians found that only 16% reported screening for social needs or domestic violence (5).

The R family's case illustrates how these social needs screeners, while a useful starting point, may fall short of addressing the problem. A simple screener for Ms. R would not have revealed the full scope of her family's needs. Even if Ms. R had successfully referred us to her father, we would not have identified the nuanced risks that were underlying this family's immediate needs. As a result, it is crucial to incorporate social needs-targeted care as a framework and set of guiding principles rather than a single task to perform. Our work with the R family began guided by a screening tool, but our goal was not to simply complete our screener or address identified needs. At every interaction, from initial screening to follow up, our goal was to give the patient the space and guidance to identify and prioritize their needs and tailor our services accordingly.

## The intervention

The Social Needs Response Team (SNRT) is a virtual team of students from medical, nursing, and social work schools that work in conjunction with and are supervised by faculty from the schools of social work, public health, and medicine. Each shift consists of interprofessional teams of 2–3 graduate level students and one licensed clinical social work supervisor.

Common SNRT referral sources include health care providers, contact tracing teams, COVID-19 community testing sites, and patients themselves. Referrals are communicated *via* healthcare electronic medical record (EMR) messages or as a voicemail on a dedicated, publicly available phone line. The team checks the EMR inbox and voicemail box at the start of each shift and individual team members connect with patients that need assistance. To review the process, each encounter begins with a member of the SNRT asking the patient what needs they would like support with. Next, the team member obtains patient consent to proceed with a more detailed and scripted questionnaire, first screening for immediate safety and distress concerns then proceeding with a detailed screener for unmet social needs. Following this, they review any identified needs with the patient, discuss what resources or services might be of assistance, and then confirm a mutually agreed upon plan and next steps. Additionally, to allow patient autonomy and decision-making in the process, SNRT members ask patients to prioritize which concerns are most important to them to address today as well as inquire if the patient would prefer to be given information about signing up for a service (such as food delivery) or would prefer the assistance of SNRT to sign up on their behalf. Often after conducting research into available resources and services, the SNRT member will call back in a designated time frame to follow up. When appropriate, a SNRT team member will then directly connect the patient to the community resource by conducting a three-way call with the community organization and the patient, or by completing any required forms for accessing services. If at any point the patient discloses safety concerns or emotional distress, we follow evidenced-based protocols for assessing and triaging domestic violence or active suicidal or homicidal ideation that results in either a warm handoff to a crisis hotline or a warm handoff to a social work supervisor (6). We provide all patients with the SNRT phone number so they can call back and reconnect for additional support at any time.

**Figure 1:**
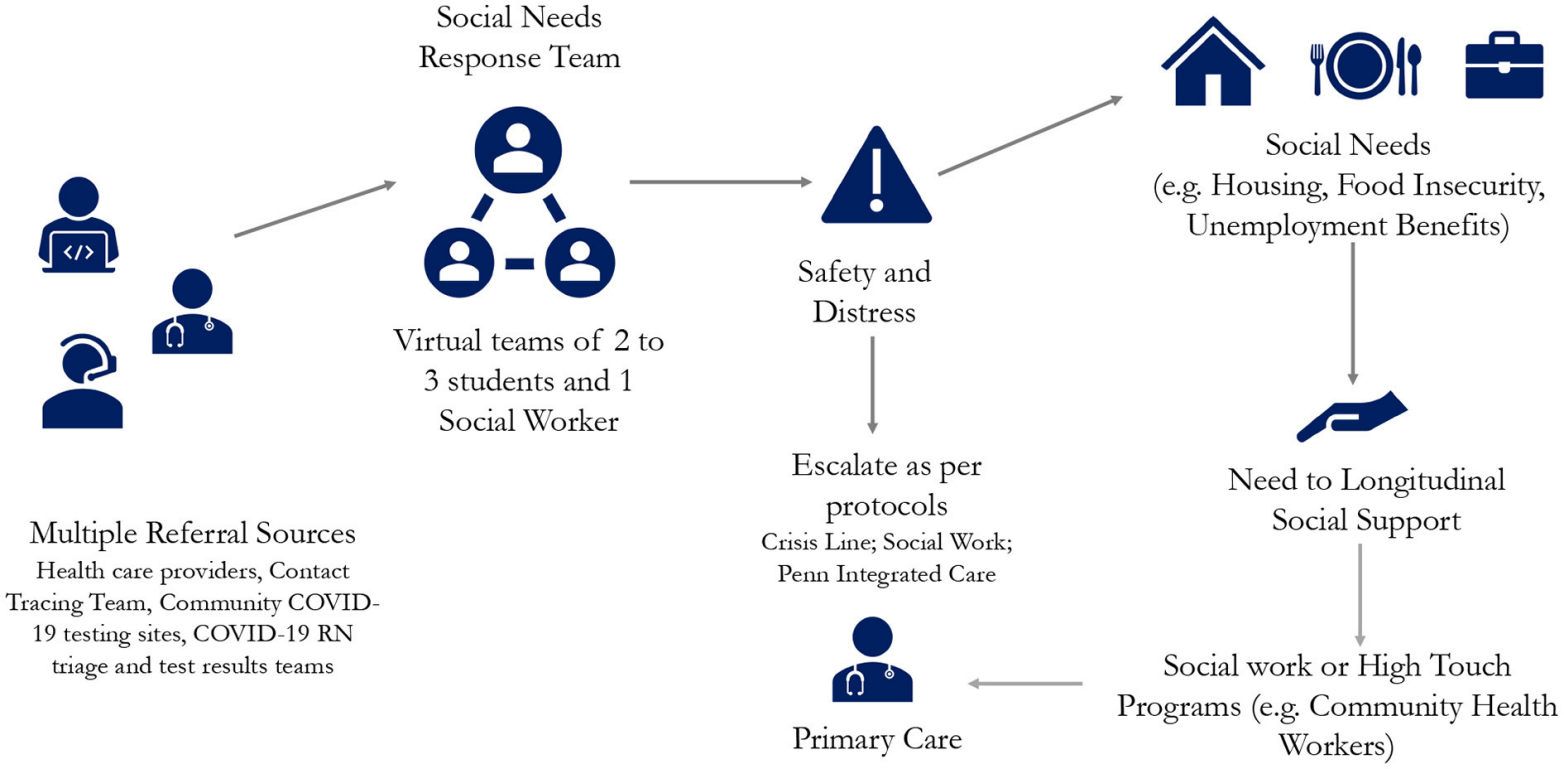
The social needs response team intervention.

The team member records each patient encounter on a templated form within the health system electronic medical records that allows for built-in data capture of discrete fields as well as free text information to facilitate follow-up with the patient and ongoing tracking of services rendered. The chart is then routed to the original referring provider and to any other health system professionals who required access to the chart, e.g., social workers or contract tracing team members (Figure 1).

**Figure 2:**
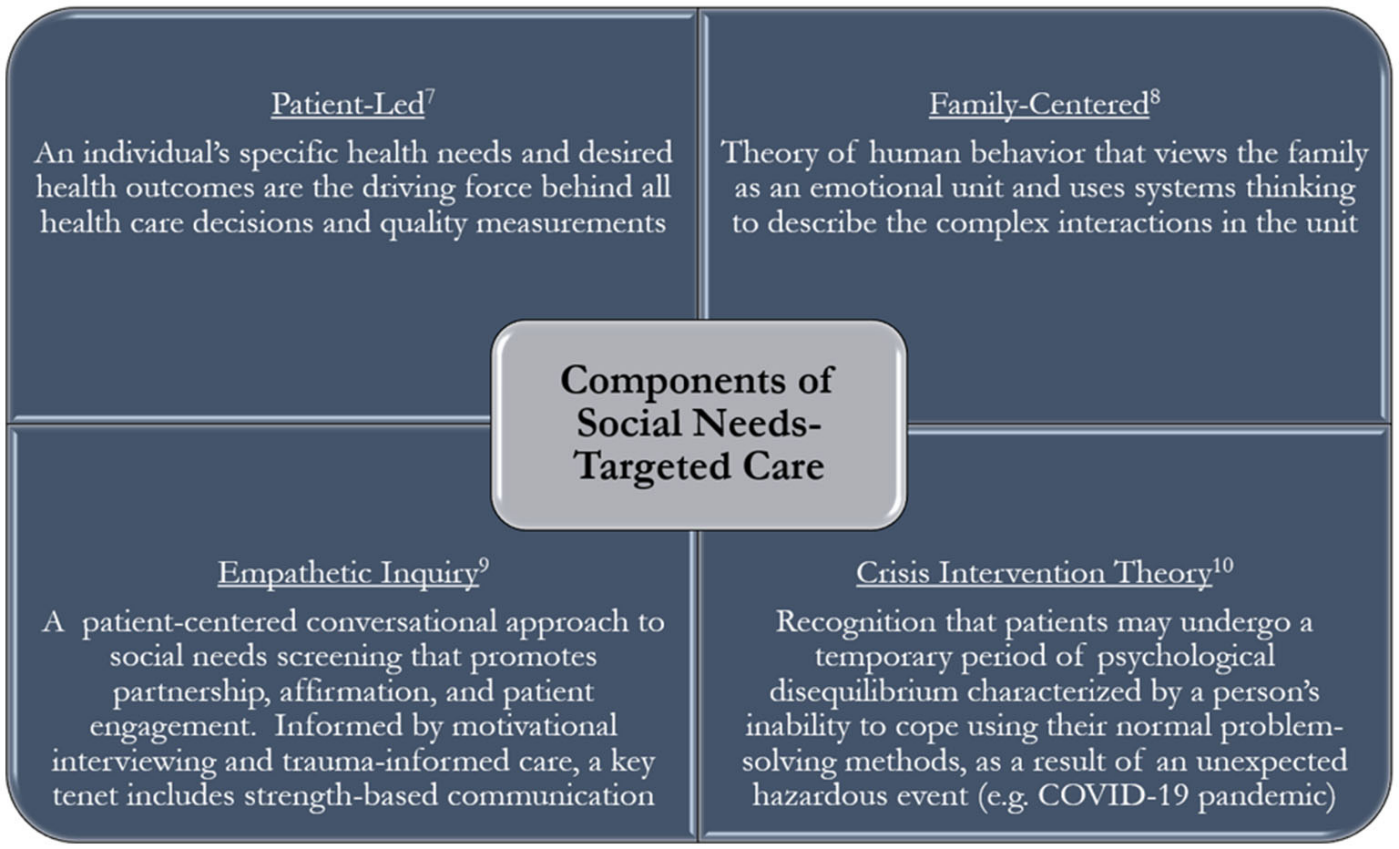
Components of social needs-targeted care.

Crucial to the tactful execution of this work is adequate training. Before engaging patients, students are trained in the tenets of crisis intervention and empathetic inquiry, as well as how to provide family-centered and patient-led care (Figure 2). Providing patient-led care dictates that patient-identified health needs and desired health outcomes are what determine the course of the conversation (7). As described above, there are multiple checkpoints in the conversation where the interviewer asks the patient to identify or prioritize their needs.

SNRT team members are also trained on what it means to provide family-centered care. We are briefed on the systems thinking theory that emphasizes the complex interactions that happen within the family unit (8). In the R family's case, it was imperative that we assessed Ms. R's home situation to identify what environmental and interpersonal needs should be addressed. If we had not asked about her household, we may have not identified that Mr. R, her father, was the primary source of income. We then would not have pursued filling out a Food Assistance application, as we could only fill one application under the head of the household's name.

SNRT members study techniques that employ empathetic inquiry; they use scripts as a guide for screening, but routinely adapt this screener and the subsequent discussion based on the patient's needs and emotional state. As mentioned, the discussion always starts by allowing the patient to inform the caller what needs are most pressing and what support we can provide. This process ensures patients who feel too overwhelmed to undergo a comprehensive screener, have an opportunity to identify their most pressing needs for the caller and receive support. Empathetic inquiry calls for a conversational approach to social needs screening that promotes partnership, affirmation, and patient engagement (9). Informed by motivational interviewing and trauma-informed care, a key tenet includes strength-based communication, which calls for understanding what strengths a patient has that can be leveraged to help address their challenges. In this case, this patient had a close-knit family and strong social supports that facilitated our ability to speak to more than one family member to better define and address their needs.

Vital to our work in a pandemic and in any situation of unmet needs, SNRT participants complete skills training on crisis intervention theory. During the COVID-19 pandemic, many patients are in the midst of a crisis—a temporary period of psychological disequilibrium, which may result in that one's inability to cope using their normal problem-solving method (10). Acknowledging that this phenomenon is well documented, team members offer to be as hands-on or hands-off in getting connected to services as the patient wishes, so that patients may get the support they need while preserving autonomy in the process. Within their structured conversations, this training empowers team members to elicit patient's needs and emotional state to dictate the conversation's length, depth, and direction. Additionally, students were trained in the importance of open-ended questioning and communication to allow interviewers to assess patient's needs in numerous ways. In the end, interviewers helped summarize patients' identified needs, supported patients in prioritizing their needs, and began offering services and solutions from which the patient could choose the best fit.

The student training in crisis intervention theory highlights Prochaska's Theory of Change, which describes various stages an individual might be in as they are considering, planning for, and/or making changes (11). It is commonly employed to assess how ready a patient may be to make changes in their life. Understanding this journey from pre-contemplative stages all the way to action and maintenance stages provides SNRT members with another tool for contextualizing the patient's current state and goals. To provide patient-centered care and preserve patient autonomy, SNRT members first identify the patient's readiness to make changes to their situation. Understanding this is part of understanding the level of support desired by the patient and allows team members to pivot their approaches based on where they find the patient.

In addition to training, mentorship plays a crucial role in ensuring high quality support is provided to patients. New students are connected to experienced students through a virtual medium during every shift, allowing for real time co-mentorship and support. The social work supervisor for every shift is available on this virtual medium to answers questions about resource eligibility or address concerns for patient safety and/or emotional distress. The ability for students and leadership to communicate in this open forum allowed participants to collaborate and continually improve upon the process. Additionally, the online messaging channels were all searchable, so if a new participant had a question about a specific patient, food delivery resources or eviction proceedings, they could search in the online messaging forum and find up-to-date information that had already been discussed.

All of this training came to the fore when providing for the R family. The family's complex social needs required a number of participants to work with them over several days. The use notes in the medical record and exchanging information online allowed new participants to get up to speed seamlessly. In applying for state benefits for the family, many questions from the Compass form arose—from what identification numbers to use to what benefits were applicable—which were answered by our social work supervisor in real time, without timing out the Compass application page.

## The outcome

After speaking with the family, the team was able to connect the family with rental assistance, the state-based Supplemental Nutrition Assistance Program (SNAP), and health insurance for one child who was uninsured at the time. The family was connected to a community organization which provided food for the duration of their quarantine period. The COVID-19 related symptoms for the family subsided and Mr. R returned to work. This interprofessional telephonic screening helped facilitate the unearthing of this family's social needs and bridged them to resources that allowed them to arrive at a socially stable situation.

As of September 2021, the Social Needs Response Team has served over 1,400 patients each with unique circumstances, strengths, and challenges as with Mr. R and his family. Approximately 43% of patients contacted spend between 30 min and 2 h on the phone with participants, a length of time not possible at most in-person visits. Over one third of patients were referred to food assistance services; another 17 and 13% were referred to housing and employment services, respectively. Approximately 45% of patients were referred to additional service providers for longitudinal support services.

## Discussion

The success of the SNRT requires a large pool of participants, an interdisciplinary leadership team committed to supervising, and a platform to allow for real time communication with leadership about patients. Though volunteer-based organizations can suffer from an inconsistent supply of participants, the SNRT has always had a steady stream of student participants. The SNRT draws from various graduate programs student participants that are eager to pick up practical skills for their future careers. Since its inception during the pandemic, the SNRT was one of few options for clinical credit available to third- and fourth-year medical students. This virtual call center program continues to satisfy educational and clinical requirements for medical students, nurse practitioner students, and social work students. Furthermore, it is now a required course for medical students participating in the primary care pathway. As such, participation in the SNRT is an integrated part of the current curricula in the University's schools of medicine, nursing, and social work.

The initiative is overseen by a dedicated faculty member from the departments of medicine and pediatrics, who meets biweekly with an interdisciplinary leadership team from the departments of social work and public health. A full-time program manager, supports this leadership team in strategic planning, data collection and analytics, daily operational decisions, curriculum development, student recruitment and training.

While the value of interdisciplinary training between students of medical, nursing, pharmacy, and social work schools is known, few programs offer opportunities for students to be active members of these interdisciplinary teams. In a 2004 review of how teamwork is taught in medical schools in the U.S., only a handful of schools focused on interdisciplinary teams. Within those schools, the training was primarily done in lecture format or small groups (12). The SNRT provides students with opportunities to actively deliver and manage care as an interdisciplinary team, rather than just learn about principles or theoretical examples of interdisciplinary care. The SNRT draws students in from medical, nursing, and social work schools as a unique opportunity to understand the vital work of interdisciplinary teams.

Though the student participant base inherently has yearly turnover, the SNRT's patient pool is similarly dynamic. Patients with ongoing needs that cannot be resolved in a matter of a few phone calls are transitioned to social work and case management. In this way, the program is not harmed by a model with natural turnover.

Finally, one of the fundamental tenets of sustainability is incentivization and, in the healthcare system, reimbursement for services. We suggest, as we detail further below, that having billable codes for social needs evaluation and management is key to the sustainability of this work–regardless of whether it is done by virtual teams or individual providers.

In addition to its participants, the work of SNRT is supported by the community-based organizations to which we referred patients and social services (like Unemployment Compensation and Pandemic Unemployment Assistance) to which we connected patients. Finally, as the pandemic and the availability of services has waxed and waned throughout the existence of the SNRT, it has been imperative to iterate on the team's methods. This iterative approach included expanding the participant base to different levels of medical students and different graduate disciplines, instituting sign-off protocols, and regularly updating the inventory of community resources to reflect changes in the community, such as the recent end to the eviction moratorium.

These partnerships have also helped to expand the reach of the SNRT. Since inception, close to 70% of referrals for patients have come from community-based contact tracing teams. These are community-based teams located in schools and Federally Quality Health Centers, amongst other locations, and ensure that our reach extends beyond those with direct contact with providers in the hospital and access to telephones. The SNRT adapted existing social work protocols to contact difficulty-to-reach patients into a robust approach taught during orientation to participating students. This approach includes but is not limited to reaching out to community-based organizations and homeless shelters to contact difficult-to-reach patients who do not have personal phones or a permanent address and has proved successful in reaching such patients to convey COVID-19 test results. Though the use of telephones is associated with multiple barriers—such as lack of a working phone or reliable connection—the SNRT's partnerships with community-based organizations and protocols for reaching patients without phones mitigates this as a barrier the equitable distribution of our services.

This case underscores the need for social needs-targeted care, a term that describes a variety of activities that aim to directly address patients' social needs (4). This family's case exemplifies the need for social needs-targeted care and has highlighted three major lessons in integrating this type of care into current healthcare systems.

*Train clinicians in social needs-targeted care and accessing available resources to address identified needs*. Strategies to promote widespread adoption of this approach include: (1) incorporating social needs-targeted care in certification exams and continuing education courses; (2) developing curricula for students and clinicians to integrate social and medical care; and (3) integrating resources with formalized processes to refer patients to community resources into existing workflows (13). Many health systems and smaller clinics have operationalized this process and have integrated it into their electronic health records.

*Reimagine care to incorporate virtual access to patients and other care professionals*. The current pandemic has called for a redefinition of care delivery. In a traditional in-person delivery method, involving multiple family members and contacting several healthcare team members from different disciplines would have taken weeks. The virtual landscape and the use of telephonic screening allows for real-time access to several members of the R family. Furthermore, the virtual format enables healthcare team members, such as the on-call social worker and student screener, to connect almost instantly. Ready access to HIPAA-appropriate text messaging and phone calls between team members facilitated dynamic and efficient decision-making. This streamlined format allowed for expeditious, yet comprehensive resolution of this family's psychosocial issues. The collaboration enabled by the virtual format allowed this intervention to serve as an interprofessional learning experience for nursing, social work, and medical students. Students worked closely with colleagues and supervisors in these other disciplines, generating the skills necessary to incorporate interprofessional social needs-targeted care in their future practices. This has resulted in a favorable outcome over just a few days.

*Incentivize clinicians to provide social needs-targeted care during routine visits*. In the United States' fee-for-service model, billing codes inputted for a given patient encounter dictate compensation for services. These may refer to disease management, preventive care, or counseling services for smoking cessation or diet. For physician's work to be compensated, there must be an applicable billing code. Most clinicians are unfamiliar with billable codes for assessment and management of social risk. These nine underutilized ICD-10-CM codes (Z55-Z65) are available to bill for assessments of psychosocial circumstances, ranging from education and housing to socioeconomic issues (14). Providers may also use CPT^®^ codes (98960-98962) to bill patient self-management of conditions as a byproduct of mitigating their social risk. In contrast to codes that bill for specific procedures or testing, the Evaluation and Management (E/M) codes, allow providers to bill for time spent managing care, with different codes for varying levels of complexity in medical-decision making. The assessment and management of social needs of a patient almost universally increases the complexity of patient care, and if coded for appropriately with these E/M codes, we could compensate providers for this additional work and time that goes into this kind of comprehensive care. While utilizing these existing codes incentivizes clinicians to practice social-needs targeted care, we also endorse the call for specific individualized codes that will enable precise capture and monitoring of social needs.

The work of SNRT, an example of a high-intensity assistance activity, is only a small step toward addressing the upstream factors found in communities that result in unmet social needs (13). This program is telephonic and maybe limited in its ability to engage patients who do not have access to a phone, although the vast majority of our patients have had access to a phone, consistent with national data on phone ownership which suggest over 95% of people have access to a mobile phone in the United States (15). SNRT team members worked tirelessly to successfully track down to assist with housing and other resources a very small set of cases of COVID-positive patients that had no home or phone, by calling local shelters. This is just one example of the challenges that underlies all efforts to mitigate social needs and that highlights the need for structural change to mitigate both the frequency and effect of unmet social needs, as detailed in a 2019 report by the National Academies of Sciences, Engineering, and Medicine. Healthcare providers and institutions can play a role in advocating for policy changes and infrastructural support that help to reduce these gaps in psychosocial needs, such as more comprehensive housing supports, equitable access to education, or more robust transportation options. Addressing these upstream factors that place certain patients at higher risk for unmet social needs could help curtail the negative effects associated with unaddressed social needs.

## Conclusion

Prior evidence reveals the long-term social, economic, and medical consequences of catastrophic events on communities and how strategies developed in crisis may be well-suited to participate in longer-term assessment and management of the community's needs (13). The pandemic revealed the resilience of healthcare professionals to find innovative ways to maintain a high standard of care for all in face of increasing barriers. Studies of frontline provider attitudes in areas hard-hit by pandemic demonstrate that providers report greater satisfaction when they are also able to address patient's emotional and social needs (16, 17). Crises like this often force us to reinvent how we deliver care—whether we are serving communities siloed in their homes due to a pandemic or devastated by natural disasters. As in the case of the SNRT, we often find that our innovations address not only these acute challenges but problems of care access and delivery that preceded the latest crisis (18). Social-needs targeted care is a longitudinal framework and should outlast any crisis. As we manage the pandemic fallout and beyond, we have a responsibility as clinicians to provide the social-needs targeted care described here, using technologically and socially innovative methods.

## Data availability statement

The original contributions presented in the study are included in the article/supplementary material, further inquiries can be directed to the corresponding author.

## Author contributions

IM and MM were both directly involved in the management of the case study described, contributed equally to the writing of the first draft of the manuscript, and contributed to describing initial implications of the case study. JA contributed to manuscript flow, added further context for the case study, and provided supervisory input throughout both the case study experience and writing experience. All authors contributed to manuscript revision, read, and approved the submitted version.

## Funding

We would like to thank the Center for Health Equity Advancement at Penn Medicine for supporting our work and providing funding to make this work an open access publication.

## Conflict of interest

The authors declare that the research was conducted in the absence of any commercial or financial relationships that could be construed as a potential conflict of interest.

## Publisher's note

All claims expressed in this article are solely those of the authors and do not necessarily represent those of their affiliated organizations, or those of the publisher, the editors and the reviewers. Any product that may be evaluated in this article, or claim that may be made by its manufacturer, is not guaranteed or endorsed by the publisher.
